# Economic costs of outbreaks of acute viral gastroenteritis due to norovirus in Catalonia (Spain), 2010–2011

**DOI:** 10.1186/s12889-015-2289-x

**Published:** 2015-10-01

**Authors:** Encarna Navas, Nuria Torner, Sonia Broner, Pere Godoy, Ana Martínez, Rosa Bartolomé, Angela Domínguez, the Working Group for the Study of Outbreaks of Acute Gastroenteritis in Catalonia

**Affiliations:** Public Health Agency of Catalonia, Department of Health, Generalitat of Catalonia, Barcelona, Spain; Public Health Department, University of Barcelona, Barcelona, Spain; CIBER Epidemiology and Public Health (CIBERESP), Institute of Health Carlos III, Madrid, Spain; Microbiology Department, Hospital Vall d’Hebrón, Barcelona, Spain

## Abstract

**Background:**

To determine the direct and indirect costs of outbreaks of acute viral gastroenteritis (AVG) due to norovirus in closed institutions (hospitals, social health centers or nursing homes) and the community in Catalonia in 2010–11.

**Methods:**

Information on outbreaks were gathered from the reports made by epidemiological surveillance units. Direct costs (medical visits, hospital stays, drug treatment, sample processing, transport, diagnostic tests, monitoring and control of the outbreaks investigated) and indirect costs (lost productivity due to work absenteeism, caregivers time and working hours lost due to medical visits) were calculated.

**Results:**

Twenty-seven outbreaks affecting 816 people in closed institutions and 74 outbreaks affecting 1,940 people in the community were detected. The direct and indirect costs of outbreaks were € 131,997.36 (€ 4,888.79 per outbreak) in closed institutions and € 260,557.16 (€ 3,521.04 per outbreak) in community outbreaks. The cost per case was € 161.76 in outbreaks in closed institutions and € 134.31 in community outbreaks. The main costs were surveillance unit monitoring (€ 116,652.93), laboratory diagnoses (€ 119,950.95), transport of samples (€ 69,970.90), medical visits (€ 25,250.50) and hospitalization (€ 13,400.00).

**Conclusions:**

The cost of outbreaks of acute viral gastroenteritis due to norovirus obtained in this study was influenced by the number of people affected and the severity of the outbreak, which determined hospitalizations and work absenteeism. Urgent reporting of outbreaks would allow the implementation of control measures that could reduce the numbers affected and the duration of the illness and thus the costs derived from them.

## Background

Acute viral gastroenteritis (AVG) is a major cause of morbidity in developed countries, with caliciviruses, and especially norovirus, being the most-frequent causal agents.

In Catalonia, norovirus is responsible for 33.3 % of outbreaks of gastroenteritis, affecting more than 1,000 people every year although the hospitalization rate is low [1, 2]. However, there is evidence that hospitalizations and deaths due to AVG are not very frequent but appear to be increasing, with 799 to 3,410 admissions attributed to norovirus in the seasons with the lowest and highest norovirus activity, respectively in England during the years 2000 to 2006 [3]. In the United States, sporadic and outbreak-associated norovirus is estimated to cause around 800 deaths and 70,000 hospitalizations each year, increasing during epidemic years associated with emergent strains and with advancing age [4] and, especially, long term health-care settings [5].

In Catalonia, the number of outbreaks of acute gastroenteritis (AG) has gradually declined in recent years from 222 in 2005 to 111 in 2009, probably because of the reduction in the number of outbreaks caused by *Salmonella spp* [6]. Likewise, there were 57 outbreaks due to norovirus in 2005 in comparison to 34 in 2009 yet the incidence rates of cases associated with outbreaks remained high (18.4 per 100,000 persons/year in 2005 to 31.7 per 100,000 person years in 2009) [2].

Studies have estimated the cost of AVG as € 16 million in Malta, €345 million in the Netherlands and A$ 342.8 million in Australia [7] (€240.13 million). The cost of outbreaks varies widely from $ 40,675 (€30,178.71) [8] to £1,200,000 (€1,514,921.67) [9] according to the number of cases and their severity and according to the different components measured and the perspective used.

Although AVG has a benign course, it generates a significant consumption of health resources. To date, there are no data on the economic impact of AVG in Catalonia and the resources used by the Public Health Agency of Catalonia in monitoring and controlling of outbreaks of AVG is unknown.

The aim of this study was to determine the direct and indirect costs of outbreaks of AVG due to norovirus in closed institutions (hospitals, social health centers and nursing homes) and the community in 2010–2011 in Catalonia.

## Methods

In Catalonia, epidemiological surveillance units (ESU) of the Public Health Agency of Catalonia are responsible for the control and monitoring of outbreaks of AVG. When an outbreak is reported to the ESU, monitoring, treatment and control of the outbreak is initiated. Stool samples are taken and sent for analysis to the microbiology laboratory of the Vall d’Hebron hospital in Barcelona.

Acute gastroenteritis was defined as diarrheal disease of rapid onset often accompanied by nausea, vomiting, fever, or abdominal pain. Outbreaks of AG were defined as AG affecting two or more persons who had similar clinical signs and symptoms, and had the same exposure to a common source of infection or by person to person transmission, or were epidemiologically linked. Epidemiological surveillance questionnaire is completed to carry out the investigation of the outbreak. Variables included in the questionnaires were demographic and clinical variables of cases such as gender, age, onset and duration of symptoms, medical consultation and treatment, occupation and need for work leave; as well as virological information obtained from stool sampling and analysis at the microbiology laboratory. Stool samples were taken from patients, workers and food handlers. Stool samples were pre-screened using standard microbiological tests to rule out bacteria and parasites and stored at −20 C Enzyme immunoassay (EIA) were performed on stool specimens. Additionally, reverse transcriptase-polymerase chain reaction (RT-PCR) detection was performed according to standard guidelines. Norovirus genotyping was performed by sequencing the amplimers obtained with the JV12/JV13 pair of primers. In outbreaks where less than three samples were collected, Kaplan’s criteria were followed [10].

All data collected during the study period was assessed. Although some data regarding food handlers, such as age, exposure to suspected food source of infection and previous AG was not always available it wasn’t considered relevant for the cost analysis.

To determine the cost and resource consumption generated by types of outbreaks we analyzed outbreaks in closed institutions (hospitals, nursing homes and social health centers) and community outbreaks. Community outbreaks include all other community settings such as school, restaurants and catering facilities.

### Direct costs

Direct costs were those that are closely related to the disease. We differentiated between direct healthcare and non-healthcare costs. We calculated the cost of outbreaks of AVG due to norovirus using information obtained from the reports made by the ESU from January 1st 2010 to December 31st 2011 as to the following variables: number of medical visits, hospital stays, drug treatment, sample processing, transport, diagnostic tests, monitoring and control of the outbreaks investigated to calculate direct costs of the outbreaks

#### Direct healthcare costs

Including the cost of medical visits, drug treatment and hospitalizations.

#### Cost of medical visits

This was calculated by multiplying the number of medical visits made (patients seen in primary care centers, in hospital emergency rooms, subsequent hospital visits, medical visits in the institution where the outbreak occurred, and private medical visits) by the unit cost of each type of visit (€ 60, €118, € 51, € 30 and €70, respectively). The weighted mean cost was calculated according to the formula:

$$ \mathrm{Weighted}\ \mathrm{mean} = {\displaystyle \sum \mathrm{w}\mathrm{x}}/{\displaystyle \sum \mathrm{w}} $$

Σ: the sum
w: the weights
x: the value

And the resulting cost to apply per visit was €61.85 in outbreaks in closed institutions and €56.93 in community outbreaks. This cost was obtained considering that patients affected by outbreaks in closed institutions made a total of 45 visits and those affected by community outbreaks made 401 visits.

#### Cost of drug treatment

We differentiated between the cost of supportive drug treatment and the cost of antibiotics. The number of patients receiving each drug prescribed recorded in the ESU reports was multiplied by the unit cost (retail price). Support drugs prescribed were: antipyretics, anti-emetics, intravenous fluids, and others.

#### Cost of hospitalization

Hospitalization costs were calculated by multiplying the number of people admitted to hospital by the number of hospital days (2.5 days for outbreaks in closed institutions and 2 days in community outbreaks) and by €536 per day of stay.

#### Direct non-healthcare costs

These included the costs of laboratory diagnosis, ESU monitoring and control, patient travel expense for medical assistance, and the transport of samples.

#### Laboratory diagnosis

Bacterial cultures and molecular techniques (RT-PCR) and were performed for all samples. Both tests were evaluated using the unit cost of the Vall d’Hebron hospital (€66.25 for PCR and €46.38 for bacterial culture).

#### Cost of epidemiological surveillance unit monitoring and control

The cost of ESU monitoring and control of outbreaks was calculated considering the time spent by technicians per outbreak and the cost per hour of the technicians (€ 15.15/hour), was calculated according to the salary table for level 22–23 civil service employees of the Generalitat of Catalonia (monthly salary of €2,499.30), and hours worked (7.30 hours/day, 22 days/month).

#### Cost of travel of cases

The number of persons who travelled for medical assistance was multiplied by the distance in kilometers for a round trip and by €0.30/km (travel rate for Government of Catalonia staff). Cases in closed institutions travelled 2.7 km, with a cost per case of €0.81, and cases in the community travelled 6 km, with a cost of €1.81 per case.

#### Cost of transport of samples

Samples were collected by physicians attending cases and sent to the reference laboratory at the Hospital Vall d'Hebron in Barcelona. The cost of transporting a sample was obtained from the mean cost of immediate special delivery courier services in 2010 and 2011, resulting in a cost of €65.70 per sample in each of type of outbreak.

### Calculation of indirect costs

Indirect costs refer to the impact of the disease in patients regarding work absenteeism, incapacity for normal activities, such as school absence, and to the time spent by families caring for patients, and the time used by patients to attend medical visits. Indirect costs were calculated with lost productivity due to work absenteeism, caregivers time and working hours lost due to medical visits.

#### Work absenteeism

This was calculated using the human capital method, which considers the value of days lost to absenteeism, equal to the value of lost productivity. Two elements were considered: days off work and the gross average inter-professional salary (€2,037.40 per month in 2011, representing a cost of €92.60/day).

#### Cost of time of children aged less than 14 years

The cost of school absence due to AVG in children aged < 14 was calculated by multiplying the days of absence by the cost per day according to the 2011 minimum wage (€ 641.40 per month).

#### Cost of time of informal caregivers

The ESU reports did not contain information on the number of days families spent caring for family members affected by AVG. Therefore, we very conservatively attributed 4 h daily to this care. The cost per hour was based on the minimum wage (€641.40 per month in 2011), and yielded a cost of €21.25 day.

All costs are expressed in 2011 Euros. The study was carried out from the perspectives of the society and the service provider. No discount rate was applied. To determine the consistency of the estimates used, we made a sensitivity analysis in which the following variables were increased or decreased by 25 %: mean salary of caregivers, time of follow up of AVG outbreak, days of work or school absence in adults and children, cots of support treatment, cost of hospitalization, length of hospital stay and number of diagnostic tests.

Data may be available upon request to the authors. The authors did not need any authorization to analyse data originated from outbreaks investigation by public health epidemiologist. The information on outbreaks gathered from the reports made by epidemiological surveillance units belongs to our institution (Public Health Agency of Catalonia).

## Results

In 2010–2011, 101 outbreaks of AVG due to norovirus were investigated in Catalonia, 66 in 2010 and 35 in 2011 (Table 1). Of these, 27 occurred in closed institutions and 74 in the community. A total of 2756 people were affected, 816 in closed institutions (29.6 %, mean of 30.22 people per outbreak; range 2–91) and 1,940 persons in community outbreaks (70.4 %, mean of 26.22 people per outbreak; range 3–191). The annual incidence rate was 18.3 per 100,000 population. No immunocompromised patients were reported.

**Table 1:** Number of outbreaks and cases of acute viral gastroenteritis due to norovirus according to year. Catalonia, 2010-2011

Year	2010	2011	Total
	*n* (%)	*n* (%)	
Number of outbreaks	66 (65.3 %)	35 (34.6 %)	101
Number of cases	1,969 (71.4 %)	787 (38.6 %)	2,756
Mean number of cases per outbreak	30.6	27.3	27.3
Range	2–191	3–91	2–191

We identified, quantified and evaluated resource consumption for the two types of outbreak. Table 2 shows the cost components evaluated and the unit costs of each component according to the ESU reports. In cases where the information was not available, it was estimated according to the specifications listed below.

**Table 2:** Cost of outbreaks of acute viral gastroenteritis due to norovirus by components. Catalonia, 2010–2011

Item	Closed institutional setting	%	Community setting	%	Weighted mean cost €	Rates
Number of consultations	45	5.5	401	20.67	56.35/56.65 ^a^	0.16 consultation/case/year
Hospitalizations	2	0.245	10	0.52	536	4.35 admissions/1000 cases/year
Treatment prescribed	267	32.7	185	9.5	3/3.01 ^a^	16.4 % of cases
Antibiotics	4	0.50	17	0.83	3.34/2.95 ^a^	0.76 % of cases
Laboratory diagnosis						
Polymerase chain reaction	476	58.13	589	30.36	66.25	38.64 % of cases
Bacteriology culture	476	58.13	589	30.36	46.38	38.64 % of cases
Transport of samples	476	58.13	589	30.36	66.70	38.64 % of cases
Surveillance unit time	76.25 h/outbreak	----	€ 15.15/h	-----	38.22/44.06 ^a^	
Transportation of cases	35 (1.2 Km/displacement)	---	409 (3 Km/displacement)	----	0.3 €/Km	0.16 displacement/case
Work absenteeism	80	9.8	142	7.32	€ 92.60	0.08 days/case
School absenteeism	-----	-----	231	11.9	21.25	0.084 days/case
Caregiver needs (days)	80 (4 h)	4.9	373 (4 h)	9.6	21.25	0.082 days/case
Transport time	35 displacements (2 h consultation)	-----	409 displacements/(2 h consultation)	-----	€11.317/h	-----
Total number of cases	816	29.6	1,940	70.4	2,756	18.3×10^3^/cases/year

^a^First value corresponds to closed setting and the second to community setting

Table 2 shows the cost components of outbreaks of AVG due to norovirus in 2010–2011. Those affected by outbreaks in this period generated 446 medical visits, a rate of 0.16 visits per year per case. Twelve patients required a total of 25 hospital days, representing 2.1 days per admission and 0.166 days per 100,000 population. A total of 452 (16.4 %) patients required supportive drug treatment and 0.76 % required antibiotics. A total of 1,065 samples were processed by RT-PCR and culture determinations. These two components were applied to 38.64 % of the patients and were transported by courier service for laboratory analysis. The cost was calculated according to the number of samples obtained in each group (476 in institutionalized patients and 589 in community outbreaks).

The cost per case of ESU monitoring and control of outbreaks was €38.12 in institutional outbreaks and €44.06 in community outbreaks. There were 222 days of work absenteeism, 231 days of school absenteeism and 227 days of informal care, a rate per case of 0.081, 0.084, and 0.082, respectively.

The weighted mean cost was €3.00 per patient in outbreaks in closed institutions and € 3.01 in patients in community outbreaks. The same procedure was used to calculate the cost of antibiotics. The antibiotics prescribed were amoxicillin and amoxicillin plus clavulanic acid). The weighted mean cost obtained was €3.34 per patient in closed institution outbreaks and € 2.95 per patient in community outbreaks.

Table 3 shows the cost of outbreaks of AVG due to norovirus in Catalonia in 2010–2011. Direct costs, which accounted for 88.50 % of total costs, included ESU monitoring (€116,652.93; 29.72 %), laboratory diagnosis (€119,950.95; 30.56 %), transport of samples (€69,970.90; 17.82 %), medical visits (€25,250.50; 6.43 %) and hospitalization (€13,400.00; 3.41 %). Indirect costs accounted for 11.50 % of total costs and included lost productivity due to work absenteeism (5.24 %) and the cost of time spent on visits 2.56 %.

**Table 3:** Cost of outbreaks of acute viral gastroenteritis due to norovirus. Catalonia, 2010–2011

	Closed institutional setting €	Community setting €	Total €	%
Consultations	2,536	22,714.50	25,250.50	6.43
Treatment	814.50	607.94	1,422.44	0.36
Hospitalization	2,680.00	10,720.00	13,400.00	3.41
Laboratory diagnosis	53,611.88	66,339.07	119,950.95	30.56
Direct healthcare costs	59,642.38	100,381.51	160,023.89	40.76
Surveillance unit follow up	31,184.45	85,468.49	116,652.93	29.72
Transportation of cases	25.20	739.98	765.18	0.19
Transportation of samples	31,273.38	38,697.52	69,970.90	17.82
Direct non healthcare costs	62,483.03	124,905.99	187,389.02	47.74
Total direct costs	122,125.41	225,287.50	347,412.91	88.50
School absenteeism	0.00	4,908.75	4,908.75	1.25
Work absenteeism	7,408.00	13,149.20	20,557.20	5.24
Caregivers time	1,700.00	7,926.25	9, 626.25	2.45
Consultation time	763.95	9,285.47	10,049.42	2.56
Total indirect costs	9,871.95	35,269.67	45,141.61	11.50
Direct & indirect costs	131,997.36	260,557.16	392,554.52	100
Cost/outbreak	4,888.79	3,521.04	3,886.68	----
Cost/case	161.76	134.31	142.44	----

The direct and indirect costs of outbreaks in closed institutions were €131,997.36 (33.63 %) and community outbreaks €260,557.16 (66.37 %). The cost per outbreak in closed institutions and the community was €4,888.79 and € 3521.04, respectively. The cost per case was €161.76 in outbreaks in closed institutions and €134.31 in community outbreaks (12.7 % lower).

The cost of ESU monitoring and control of outbreaks was calculated considering the time spent by technicians per outbreak (76.15 hours/outbreak).

During the study period there were two deaths in elderly people aged 90 and 101 years, respectively, with AVG, but due to comorbidities, deaths were not attributed to AVG and therefore the cost per death were not calculated.

The sensitivity analysis shows that modifying the study variables produced only small variations in the cost per outbreak and therefore the results of the analysis for the base case were consistent (Table 4).

**Table 4:** Sensitivity analysis

Sensitivity analysis (SA): AVG community outbreaks					Sensitivity analysis (SA): AVG outbreaks in closed institutions							
BASE CASE: Cost of outbreak €3,521.04; Cost of Case €134.31							BASE CASE: Cost of outbreak €4,888.79, Cost of case €161.76					
Sensitivity analysis type	Outbreak cost	Variation		Case cost	Variation		Outbreak cost	Variation		Case cost	Variation	
		Amount €	%		Amount €	%		Amount €	%		Amount €	%
Average salary caregiver: Δ25% (€ 92. 60)	3,880.69	359.64	10.21	148.03	13.72	10.22	5,100.2	211.41	4.32	168.76	7	4.33
Average salary caregiver: (€ 0. 00)	3,413.93	−107.11	−3.04	130.22	−4.09	−3.05	4,825.83	−62.96	−1.29	159.68	−2.08	−1.29
ESU Follow up: ∇25 % (€ 57.19)	3,232.34	−288.70	−8.20	123.30	−11.01	−8.20	44,600.08	−288.71	−5.91	152.21	−9.55	−5.90
Follow EUV outbreak Time: Δ25% (€ 95.31)	3,808.75	287.71	8.17	145.32	11.01	8.20	5,116.91	228.12	4.67	169.31	7.55	4.67
Days down children: Δ 25 % (288.75 days)	3,554.21	33.17	0.94	135.57	1.26	0.94	4,973.12	84.33	1.72	164.55	2.79	1.72
Days off work adults: Δ25% (177.5 days)												
Low children days: Δ 25 % (173.25 days)	3,466.43	−54.61	−1.55	132.22	−2.09	−1.56	4,804.46	−84.33	−1.72	158.97	−2.79	−1.72
Days off work adults: Δ25% (106.5 days)												
Treatment cost: Δ25% (€759.9 community & €1018.12 closed institutions outbreaks)	3,523.10	2.06	0.06	134.39	0.08	0.06	4,896.33	7.54	0.15	162.01	0.25	0.15
Stay cost: Δ25% (€670) children & adults	3,557.26	36.22	1.03	135.69	1.38	1.03	4,913.61	24.82	0.51	162.58	0.82	0.51
Stay cost: ∇ 25 % (€402) children $ adults	3,484.83	−36.21	−1.03	132.93	−1.38	−1.03	4,867.31	−21.48	−0.44	161.05	−0.71	−0.44
Days of stay: Δ 25 % (2.5 days) children& adults	3,557.26	36.22	1.03	135.69	1.38	1.03	4,913.61	24.82	0.51	162.58	0.82	0.51
Days of stay: ∇ 25 % (1.75 days) adults & children	3,502.93	−18.11	−0.51	133.62	−0.69	−0.51	4,863.98	−24.81	−0.51	160.94	−0.82	−0.51
Stay 2 day:Δ100% (4 days)	3,665.91	144,87	4.11	139.83	5.52	4.11	4,988.05	99.26	2.03	165.05	3.29	2.03
Diagnostic tests: Δ25% (€736,25)	3,875.90	354.86	10.08	147.84	13.53	10.07	5,674.77	785.98	16.08	187.77	26.01	16.08
Diagnostic tests: ∇25 % (€441,75)	3,166.19	−354.85	−10.08	120.77	−13.54	−10.08	4,102.82	−785.97	−16.08	135.75	−26.01	−16.08

## Discussion

In 2010–2011, most outbreaks of AGV due to norovirus (74 % of outbreaks and 70.4 % of cases) occurred in the community. However, the cost per outbreak in the community (€134.31) was 12.7 % lower than the cost of outbreaks in closed institutions (€161.76), due to the greater severity, and therefore greater use of resources, of cases in closed institutions. The proportion of outbreaks occurring in closed institutions varies between the 71 % found by Fretz et al. [11] in Switzerland, the 39 % found by Blanton et al. [12] in the United States, the 72 % observed by Hellard et al. [7] in Australia, and the 82 % found by Van den Brandhof in Holland [13]. In Catalonia, the cost of AVG outbreaks in 2010–2011 was €392,554.52. Direct costs accounted for 88.50 % of the total: medical visits, days of hospital stay and drug treatment accounted for 10.20 %, laboratory diagnoses for 48.38 % and the ESU for 29.72 %. Indirect costs accounted for the remaining 11.50 % of the total cost due to lost productivity from time off work or school of cases. Gauci et al. [14] found that direct costs accounted for 70.25 % of the cost of outbreaks of infectious intestinal disease in Maltese community, while Dalton et al. [15] found direct costs accounted for 92 % of the total in a study in food handlers in Denver (Colorado, USA) and Lucioni et al. [16] found direct costs were 78 % of the total in a study of the consumption of contaminated food in Puglia (Italy). Other studies of outbreaks of AVG have found a lower proportion attributable to direct costs, such as the 18 % found by Van den Brandhof et al. [13] and the 22 % observed by Hellard et al. [7] with a restriction of normal activity occurring in 52 % of cases.

When comparing the result obtained in our study in which the cost per case in the community outbreaks is of €134,31, with other studies, there is a great variability. In the study of Henson et al. [17] "Gastrointestinal Estimation of the costs of acute illness in British Columbia, Canada” carried out between June 2002 and June 2003, the cost per case was 2,170.20 €, while in the study of Gauci et al. [14] it was 103,52 €. In the study of Van de Brandhof et al. [13] carried out in Holland, the cost per case of AVG was of €77 and in the community study carried out by Hellard et al. [7] in Australia €14,27. The variability of the results, not taking into account differences of costs of each health system and of occupational loss of productivity that can exist among different countries, is motivated fundamentally by the severity of the symptoms and the number of days lost of work of cases.

In our study, the number of days of lost productivity was 0.08 days per case, school absenteeism was 0.084 days per case and days of care were 0.082. Gauci et al. found similar results: 0.26 days of lost productivity, 0.0784 days of school absenteeism and 0.0045 days of care [14].

The hospitalization rate in our study was 0.44 %, similar to the rate reported by Van den Brandhof et al. (0,5 %) [13] but higher than the rate reported by Hellard et al. (0.0816 %) [7] and by Henson et al. (0.003 %) [17]. In contrast, Gauci et al. found that as much as 6.35 % of cases were hospitalized [14].

In our study, direct costs included diagnostic tests and the transport of samples for analysis. These two components were applied to 38.64 % of patients, accounting for 48.38 % of the total cost, whereas in other studies diagnostic test were prescribed in only 4.46 % [7] and 5.40 % [14] of cases. Given the high cost of these components (30.56 % of direct costs on laboratory diagnosis and 17.82 % on transport of samples to the reference laboratory for analysis), it seems appropriate to search for cheaper alternatives such as availability of laboratory tests in a wide range of clinical laboratories, not only public health facilities.

To reduce the costs it would be convenient that cases were reported as soon as they are detected, to avoid growing number of cases. Moreover in closed institutions, strict hygiene and preventive guidelines should be followed to avoid onset of new cases. Another aspect that would reduce the cost of outbreaks would be to concentrate clinical samples for transport and processing them all together. Although this can cause delay in confirmation of the causal agent and could therefore derive in the appearance of more secondary cases. In this sense it would be convenient to carry out a cost-effectiveness study to assess which alternative is more cost saving : to keep on with the current procedure of sending the samples when cases are reported or sending them in a more aggregated manner that would decrease transportation and processing costs.

We found that the cost of ESU monitoring was €900.60 per outbreak. Zingg et al. [8] estimated these costs at $ 1,408 (€1,045.67) per outbreak but did not include them in their study, arguing that these units already exist and no extra staff were needed. We included these costs in the study on the grounds that the functioning of the units has an opportunity cost, and if staff were not monitoring AVG outbreaks they could be monitoring another public health problem.

Our results show that 2,756 cases of AVG due to norovirus occurred in Catalonia in 2010–2011 [18]. Taking the population of Catalonia at this time [19], this represents a rate of cases associated to outbreaks of 18.3 per 100,000 persons/year . However, the true rate may be higher, since we only studied reported outbreaks and did not take into account sporadic cases or non-reported outbreaks. The rate is slightly lower than those of previous years ranging from 18.4 per 100,000 persons/year in 2005 to 31.7 per 100,000 persons/year in 2009 [2, 20].

There were two deaths registered in AVG cases during the study period, yet the cause of death was finally reported as unrelated to AVG disease but instead caused by pre-existing underlying diseases. Although AVG can be potentially life-threatening in immunocompromised patients, the fact that no immunocompromised patients were reported could explain the low mortality observed in our study.

As other authors have stated, we cannot rule out the possibility of unreported deaths due to AVG. Trivedi et al. in a study of nursing homes in the states of Oregon, Wisconsin and Pennsylvania in 2009–2010 found that deaths due to norovirus rose from 41.9 per residence per year when there were no outbreaks to 53.9 per residence per year when outbreaks occurred. The authors estimated an overall excess mortality of 11 % in the study population [21].

The cost per outbreak of AVG due to norovirus in Catalonia (€3,534.49) was within the lower range of that found in other studies: $ 40,675 [8] (€30,178.71), $ 657,644 [22] (€488,067.81) and £1.2 million (€1.5 million) [9]. In these studies, there were a large number of healthcare workers affected, and it was necessary to close beds in affected hospitals, a situation that did not occur in our study.

Limitations to the study include underreporting of small outbreaks and no reporting of sporadic cases; no ward closures in long-term care facilities or hospitals and, in foodborne outbreaks, only on primary and secondary cases were investigated, no information was gathered for tertiary person to person spread. Epidemic outbreaks of any kind, are under a statutory reporting condition and because norovirus AG is not a notifiable disease in Spain, as in most countries, outbreaks are the only feasible tool for research studies. These are all issues that underestimate the economic and disease burden caused by norovirus.

This study is an example of applied research. An example on how and why public health activities should be assessed. Public health performance is evaluated according to different outcomes, and the economic aspects are something that are not assessed as often as they should be. There is no doubt in considering that the information that this work brings up is best for those health professionals that work in outbreak management and surveillance, but it may also be considered informative to citizenship to learn on how part of their taxes are spent. In all, economic assessments should be carried out in a systematic manner to be aware of the economic burden a public health performance represents and be able to achieve a more efficient management of outbreaks.

## Conclusions

In conclusion, the cost of outbreaks of AVG due to norovirus was influenced by the number of cases and the severity of the outbreak, which determined hospitalizations and work absence. Urgent reporting of outbreaks to the ESU is essential in order to take rapid action to reduce the number of cases and the length of the process.

The lack of widespread availability of laboratory confirmation RT-PCR technique makes it necessary to transport case samples from place of outbreak occurrence to the public health laboratory. This fact increases outbreak investigation expense, thus norovirus testing and confirmation should be more readily available to microbiology laboratories enhancing detection of sporadic cases that are now under detected.

Health education for food handlers and outbreak control measures (such as hand washing, which has been shown to reduce episodes by 30 %) [23, 24] should be stressed in order to reduce the incidence of outbreak-related cases and in turn, their cost. Dedicating scarce resources to a specific intervention means that other actions, which might be more efficient, are not carried out. Preventive measures for outbreaks of AVG due to norovirus should be intensified, given the health and social costs they represent.

## Abbreviations

AG: Acute gastroenteritis
AVG: Acute viral gastroenteritis
€: Euros
ESU: Epidemiological surveillance units
RT- PCR: Reverse transcriptase - Polymerase chain reaction

## References

[1] Dominguez A, Torner N, Ruiz L, Martinez A, Barrabeig I, Camps N, Godoy P, Minguell S, Parron I, Pumares A, Sala MR, Bartolome R, de Perez U SM, Montava R, Buesa J (2008). Aetiology and epidemiology of viral gastroenteritis outbreaks in Catalonia (Spain) in 2004–2005. J Clin Virol.

[2] Torner N (2009). Clinical and epidemiological study of viral gastroenteritis outbreaks in Catalonia. Rev Esp Salud Publica.

[3] Haustein T, Harris JP, Pebody R, Lopman BA (2009). Hospital admissions due to norovirus in adult and elderly patients in England. Clin Infect Dis.

[4] Lopman BA, Hall AJ, Curns AT, Parashar UD (2011). Increasing rates of gastroenteritis hospital discharges in US adults and the contribution of norovirus, 1996–2007. Clin Infect Dis.

[5] Desai R, Hembree CD, Handel A, Matthews JE, Dickey BW, McDonald S, Hall AJ, Parashar UD, Leon JS, Lopman B (2012). Severe outcomes are associated with genogroup 2 genotype 4 norovirus outbreaks: a systematic literature review. Clin Infect Dis.

[6] Torner N, Dominguez A, Ruiz L, Martinez A, Bartolome R, Buesa J, Ferrer MD (2008). Acute gastroenteritis outbreaks in Catalonia, Spain: norovirus versus Salmonella. Scand J Gastroenterol.

[7] Hellard ME, Sinclair MI, Harris AH, Kirk M, Fairley CK (2003). Cost of community gastroenteritis. J Gastroenterol Hepatol.

[8] Zingg W, Colombo C, Jucker T, Bossart W, Ruef C (2005). Impact of an outbreak of norovirus infection on hospital resources. Infect Control Hosp Epidemiol.

[9] Danial J, Cepeda JA, Cameron F, Cloy K, Wishart D, Templeton KE (2011). Epidemiology and costs associated with norovirus outbreaks in NHS Lothian, Scotland 2007–2009. J Hosp Infect.

[10] Kaplan JE, Feldman R, Campbell DS, Lookabaugh C, Gary GW (1982). The frequency of a Norwalk-like pattern of illness in outbreaks of acute gastroenteritis. Am J Public Health.

[11] Fretz R, Svoboda P, Schorr D, Tanner M, Baumgartner A (2005). Risk factors for infections with Norovirus gastrointestinal illness in Switzerland. Eur J Clin Microbiol Infect Dis.

[12] Blanton LH, Adams SM, Beard RS, Wei G, Bulens SN, Widdowson MA, Glass RI, Monroe SS (2006). Molecular and epidemiologic trends of caliciviruses associated with outbreaks of acute gastroenteritis in the United States, 2000–2004. J Infect Dis.

[13] Van den Brandhof WE, de Wit GA, de Wit MA, van Duynhoven YT (2004). Costs of gastroenteritis in The Netherlands. Epidemiol Infect.

[14] Gauci C, Gilles H, O'Brien S, Mamo J, Stabile I, Ruggeri FM, Calleja N, Spiteri G (2007). Estimating the burden and cost of infectious intestinal disease in the Maltese community. Epidemiol Infect.

[15] Dalton CB, Haddix A, Hoffman RE, Mast EE (1996). The cost of a food-borne outbreak of hepatitis A in Denver, Colo. Arch Intern Med.

[16] Lucioni C, Cipriani V, Mazzi S, Panunzio M (1998). Cost of an outbreak of hepatitis A in Puglia, Italy. Pharmacoeconomics.

[17] Henson SJ, Majowicz SE, Masakure O, Sockett PN, MacDougall L, Edge VL, Thomas MK, Fyfe M, Kovacs SJ, Jones AQ (2008). Estimation of the costs of acute gastrointestinal illness in British Columbia, Canada. Int J Food Microbiol.

[18] Sala MR, Broner S, Moreno A, Arias C, Godoy P, Minguell S, Martinez A, Torner N, Bartolome R, de Simón M, Guix S, Dominguez A (2014). Cases of acute gastroenteritis due to calicivirus in outbreaks: clinical differences by age and aetiological agent. Clin Microbiol Infect.

[19] Institut d'Estadística de Catalunya IDESCAT. Padró municipal d'habitants per sexe. Xifres oficials. Catalunya. 2014. Sèrie temporal. Available at: http://www.idescat.cat/ Cited January 8, 2014.

[20] Dominguez A, Broner S, Torner N, Martinez A, Jansa JM, Alvarez J, Barrabeig I, Cayla J, Godoy P, Minguell S, Camps N, Sala MR (2010). Utility of clinical-epidemiological profiles in outbreaks of foodborne disease, Catalonia, 2002 through 2006. J Food Prot.

[21] Trivedi TK, DeSalvo T, Lee L, Palumbo A, Moll M, Curns A, Hall AJ, Patel M, Parashar UD, Lopman BA (2012). Hospitalizations and mortality associated with norovirus outbreaks in nursing homes, 2009–2010. JAMA.

[22] Johnston CP, Qiu H, Ticehurst JR, Dickson C, Rosenbaum P, Lawson P, Stokes AB, Lowenstein CJ, Kaminsky M, Cosgrove SE, Green KY, Perl TM (2007). Outbreak management and implications of a nosocomial norovirus outbreak. Clin Infect Dis.

[23] Ejemot RI, Ehiri JE, Meremikwu MM, Critchley JA. Hand washing for preventing diarrhoea. Cochrane Database Syst Rev. 2008; CD004265. 10.1002/14651858.CD004265.pub2 18254044

[24] Harris JP, Lopman BA, O'Brien SJ (2010). Infection control measures for norovirus: a systematic review of outbreaks in semi-enclosed settings. J Hosp Infect.

